# Spot-on phage therapy: stable formulations, smarter dosing for topical phage application

**DOI:** 10.3389/fcimb.2026.1697070

**Published:** 2026-04-14

**Authors:** Jagnoor Singh Sandhu, Amrita Parida

**Affiliations:** 1 Central Animal Research Facility, Kasturba Medical College, Manipal Academy of Higher Education, Manipal, India; 2 Department of Pharmacology, Kasturba Medical College, Manipal Academy of Higher Education, Manipal, India

**Keywords:** biofilms, formulation stability, moi, phage therapy, PK/PD, topical delivery

## Abstract

The growing antimicrobial resistance crisis has led to renewed interest in bacteriophage therapy, mostly for topical uses such as wound/burn care. However, the clinical application of topical phage therapy is delayed due to a major problem that dosing and delivery protocols lack standardization. By gathering scattered published studies on topical phage therapy, this review attempts to bridge the most important gap. We unpack the complicated interactions between phage titer (most of the time 10^7^–10^9^ PFU/mL), multiplicity of infection (MOI), and the stability of various formulations such as hydrogels, creams, and polymer-based sprays, which are some of the factors that determine the effectiveness of the treatment to the greatest extent. Our review extends to the bacterial load and biofilm maturity, whose raising is the main explanation of why mature biofilms need higher, repeated dosing or a combination of antibiotics and depolymerase-armed phages to be treated effectively. Additionally, we collect pharmacokinetic/pharmacodynamic (PK/PD) essentials from animal experiments and talk about the role of wound dressings in the controlled delivery of phages. The authors argue that topical phage therapy can be most effective only when it is a combined effort: accurate dose calculation, intelligent formulation design, and careful planning of the time for application. To get from the laboratory to the clinic, the field needs to urgently implement standardized PK/PD frameworks, stringent stability testing, and comprehensive clinical trials. This paper brings together these components to serve as a practical guide in the development of efficient, dependable, and easily translatable topical phage treatment regimens.

## 1 Introduction

The resurgent use of phage therapy as a viable antibiotic alternative necessitates the elaboration of precise dosage strategies especially for topical application, in which both the stability of the formulation and the delivery of the agent represent separate issues. Topical treatments are modified according to the severity of the infection and the bacterial load (Ahmad et al., 2024; Armal et al., 2025), with the Multiplicity of Infection (MOI) being a main factor that very low MOIs can promote phage replication (Wang et al., 2024a), while thus elevated MOIs lead to enhanced bacterial killing (Silva et al., 2014); nevertheless, the usage of high doses can raise the probability of resistance and other side-effects (Young et al., 2024; Aziz et al., 2025). Achieving this balance means managing several interconnected factors at same time: preserving phage concentration in the chosen vehicle (for example, cetomacrogol cream at 2.5 × 10^8^ PFU/g; Brown et al., 2016, or polymer sprays; Chang et al., 2022b), ensuring precise application through devices such as metered-dose dispensers (Liu et al., 2016), and addressing biofilm maturity since established biofilms markedly impede penetration and often call for combined approaches (Chang et al., 2022a; Sandhu et al., 2025). Pharmacokinetic and pharmacodynamic elements timing after wound debridement, whether bacteria are planktonic or biofilm-associated, and the choice of dressing all further shape outcomes; for instance, hydrogels help retain moisture and can support sustained phage activity (Mary et al., 2024). While existing literature has proved the potential of topical phage therapy and documented certain challenges such as dosing (Ahmad et al., 2024), formulation stability (Brown et al., 2016), and biofilm penetration (Chang et al., 2022a) these elements are often discussed in isolation.

While existing literature has proved the potential of topical phage therapy and documented certain challenges such as dosing (Ahmad et al., 2024), formulation stability (Brown et al., 2016), and biofilm penetration (Chang et al., 2022a) these elements are often discussed in isolation. This review critically evaluates the available scattered and complex data on topical phage therapy and compares with existing to ascertain effective concentration dosing with a suitable formulation, combines PK/PD considerations, and choosing the effective delivery methods (such as hydrogels and metered-dose devices) to counteract the effects of high bacterial loads and biofilm maturity on local phage therapy. By consolidating and critically appraising the latest translational and clinical evidence, this review aims to equip researchers and clinicians with the knowledge needed to design effective, reliable, and clinically translatable topical phage therapies, so that we can bridge the gap between promising lab results and clinical application.

## 2 Dosing considerations for topical phage therapy

Dosing for topical phage therapy must be tailored to the infection’s severity and location: typical therapeutic concentrations range from 10^7^ to 10^9^ PFU/mL and may be given as a single dose or repeatedly over days to weeks, with the choice of route topical, oral, intravenous, or intraperitoneal guided by the site and characteristics of the infection; for example, intravenous regimens of approximately 10^9^ PFU per administration have reported successful outcomes (Ahmad et al., 2024). The multiplicity of infection (MOI) is one of the major concepts which means the phage-to-bacterium ratio and it is the factor that changes the results drastically. Reported optimal MOIs vary widely: exceptionally low values (0.0001) have shown efficacy with phage titers rising to 10^10^ PFU/mL (Wang et al., 2024a), while much higher MOIs (10, 100, 1000) accelerate bacterial killing an MOI of 100 produced a 4.1 log CFU/mL reduction after 8 hours (Silva et al., 2014). To avoid the development of resistance, dose optimization should be done very carefully, because a very high phage load can lead to the creation of a selective pressure that will favor resistant variants (Young et al., 2024). Therefore, MOI and total dose should be local infection site, bacterial density, and phage replication kinetics adjusted to clear the pathogen while at the same time, there is a risk of toxicity or immune activation (Ghosh et al., 2025). Put simply, the effectiveness depends most on the relative numbers of phage and host cells, and therefore, both factors should be adjusted when determining the therapeutic dose that will be effective (Onarinde and Dixon, 2018).

### 2.1 Key considerations

**Figure 1:**
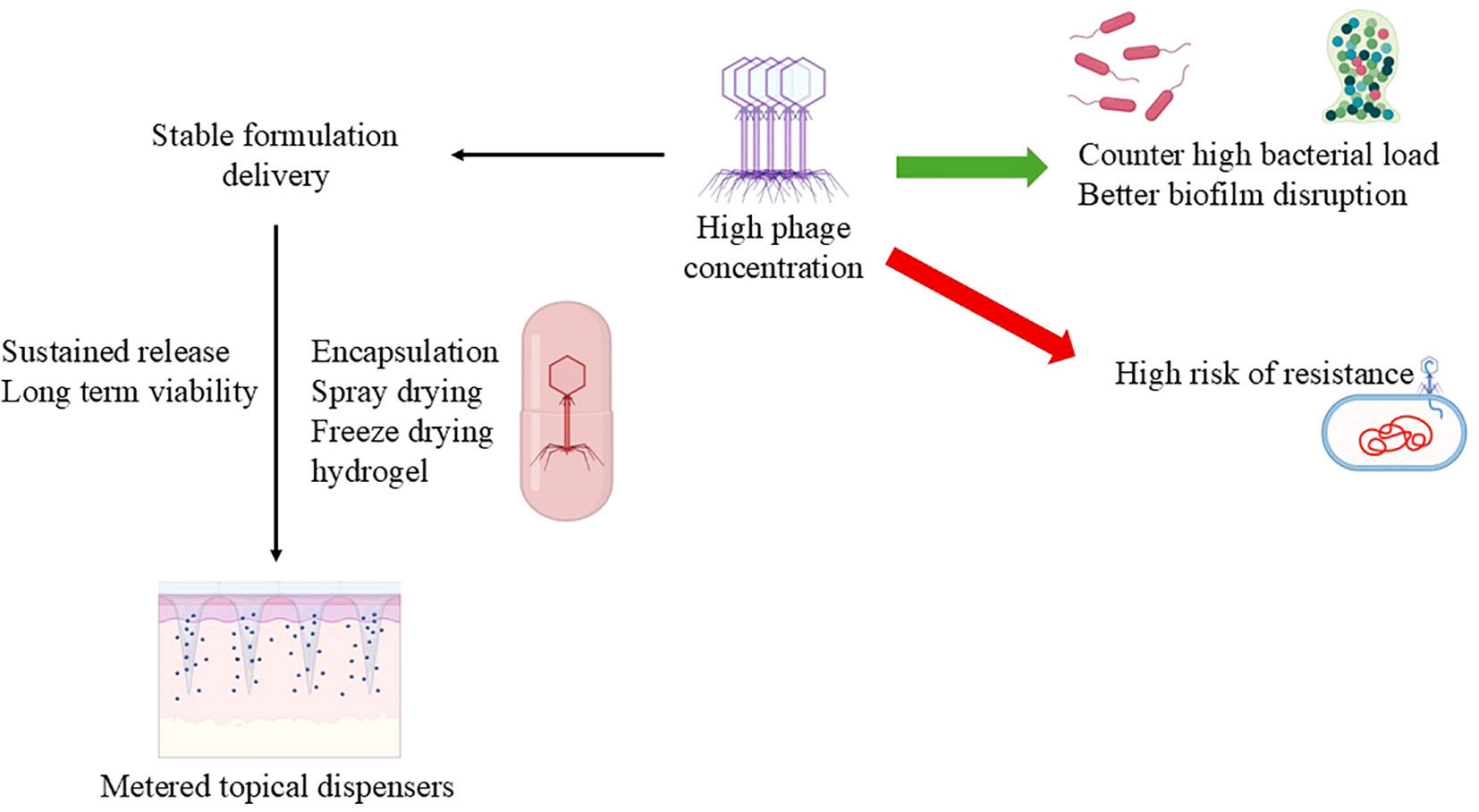
Schematic representation of phage formulation strategies.

Several factors that are tightly interrelated need to be considered in phage dosing and delivery. The amount of virus in a preparation is the most significant factor that changes the clinical effect: for instance, a cetomacrogol cream formulated at 2.5 × 10^8^ PFU/g was effective against *Propionibacterium acnes*, whereas liquid suspensions at 7 log PFU/mL (10^7^ PFU/mL) lost activity below therapeutic levels after a week of storage (Brown et al., 2016; Duyvejonck et al., 2021), and a higher phage load has been associated with more efficient biofilm disruption (Sandhu et al., 2025). Just as it is, formulation stability is of equal significance. Carriers such as cetomacrogol creams and phage–polymer sprays can sustain release and preserve viability, thus improving wound healing and bacterial clearance (Mary et al., 2024; Chang et al., 2022b). How the product is applied matters too: metered topical dispensers give consistent, precise dosing, while occlusive emollients or dressings can provide controlled, prolonged release at the wound surface (Liu et al., 2016; Mary et al., 2024). Finally, dose design must be informed by phage pharmacokinetics and pharmacodynamics host immune status, the type of infection, and the site of application all shape phage persistence and activity, so PK/PD considerations are essential when setting a regimen (Jończyk-Matysiak et al., 2019; Young et al., 2024). Details are summarized in **Table 1**, **Figure 1**.

**Table 1:** Key factors and considerations for effective topical phage therapy in wound management.

Factor	Consideration	Example/Study reference
Phage Concentration	Maintain effective concentration (e.g. 6–9 log PFU/mL)	(Brown et al., 2016; Duyvejonck et al., 2021)
Formulation Stability	Use stable formulations like cetomacrogol-based creams	(Chang et al., 2022a; Mary et al., 2024)
Application Method	Utilize metered-dose dispensers for accurate dosing	(Liu et al., 2016)
PK/PD Understanding	Consider patient-specific factors and infection site	(Jończyk-Matysiak et al., 2019; Young et al., 2024)

References correspond to specific studies demonstrating the relevance of each factor: (Brown et al., 2016; Duyvejonck et al., 2021) phage concentration optimization (Chang et al., 2022a; Mary et al., 2024), formulation stability (Liu et al., 2016), application method precision, and (Jończyk-Matysiak et al., 2019; Young et al., 2024) PK/PD understanding in clinical contexts.

### 2.2 Dose calculation

#### 2.2.1 Determine the effective concentration

Identifying the optimal concentration for the specific phage–bacteria combination is the first step. Maintaining titres of 6–9 log PFU/mL is generally effective (Duyvejonck et al., 2021). Clinical wound use often involves ~10^6^ PFU/mL, while some commercial products (e.g., the Eliava cocktail) are around 10^7^ PFU/mL (Armal et al., 2025; Chang et al., 2020). An Effective Therapeutic Titer (ETT) near 7.0 log PFU/mL after incubation with wound-care products is recommended to ensure sufficient activity (Merabishvili et al., 2017). Concentrations of 10^8–^10^9^ PFU/mL deliver superior biocontrol (Zyman et al., 2022), although excessive titres risk lysis-from-without and neutralizing antibody production (Aziz et al., 2025; Zyman et al., 2022). Efficacy also depends on bacterial load, formulation properties, and delivery mechanisms (Armal et al., 2025; Morozova et al., 2018).

#### 2.2.2 Select an appropriate formulation

Phages must exhibit efficient absorption and lytic activity against target bacteria. Temperate phages should be avoided due to virulence amplification and transduction risks (Kakasis and Panitsa, 2019). Narrow-host-range phages preserve microbiome integrity, though broad strain coverage within target species is advantageous (Abd-Allah et al., 2022).

The process of wound healing involves different stages and is very complicated. Indurated wounds are best treated with the help of chitosan, which is a substance that has been proven to be bactericidal, anti-inflammatory, and hemostatic in nature. Sikora et al. (2024) examined the preparation and performance of chitosan-based films with the incorporation of phages lytic to *Pseudomonas* (KTN4, KT28, and LUZ19) to obtain an antibacterial wound dressing that would help wound healing. Besides that, the research also looked into the method of synthesizing a polymer from microcrystalline chitosan (MKCh) as a matrix for phage deposition. The findings confirmed that the phage particles had bonds with chitosan polysaccharide and thus they were trapped in the material matrix. Nevertheless, as a result of the material being highly hydrophilic and having a large swelling capacity, bacterial culture medium can penetrate the matrix, hence the embedded phages can directly interact with bacteria and cause their lysis.

Electrosprayed phage-loaded lactose particles appear to be a potential method for the treatment of bacteria causing oral and outer-ear infections. The researchers made a solid formulation by dissolving 40% w/v total solute (lactose monohydrate: polyvinyl alcohol = 9:1 w/w) in SM buffer with 0.1% (v/v) Triton X-100, then added phage to a final concentration of 50% (v/v). Spherical particles with an encapsulation efficiency of 31 ± 8% were produced by electrospraying this mixture. Liu et al. (2025) indicate that these phage-loaded particles kept losing their viability gradually during storage, and the reductions at room temperature and 4°C were more significant than those for the unformulated phage stock. Cetomacrogol cream is a hydrophilic, non-greasy, and non-ionic formulation that contains a phosphate buffer at pH 5.5. This buffer ensures that the active ingredients sensitive to higher pH levels remain stable. The non-ionic cream base, by its nature, lowers the interactions between ions and phage particles, thus the chance of phage deactivation is kept low (Jończyk-Matysiak et al., 2017; Piranaghl et al., 2023).

Phages in cetomacrogol cream have shown good stability, being capable of retaining their activity for up to 90 days when stored at 4°C in the dark, with only approximately a 1-log titer reduction. Nevertheless, the phage inactivation was observed within 14–21 days as a result of the exposure of phages to high temperatures (45°C) or full light at room temperature (20–25°C) (Jończyk-Matysiak et al., 2017; Chang et al., 2020). Stable, controlled-release formulations such as cetomacrogol creams and non-ionic polymer sprays are still on the way to becoming a significant source of clinical applications for therapeutic purposes (Brown et al., 2016; Chang et al., 2022a; Mary et al., 2024). Encapsulation strategies stabilize phages against environmental stressors while enabling practical administration (Samananda Singh, 2024), improving protection and prolonging topical activity (Veverka et al., 2020). Polymer/lipid coatings mitigate processing and storage stresses (Malik et al., 2017). A study by Piranaghl et al. (2023) illustrates the application of polyethylene glycol (PEG)-based ointments as a medium for bacteriophages to localize the treatment of *Pseudomonas aeruginosa*-infected burn wounds in mice. In particular, the scientists prepared a topical application containing phages in a PEG 400/PEG 4000 (8:4 g) semi-solid base. Their results showed that the phages were still active and stable in the PEG ointment. Moreover, the article points out that non-ionic PEG carriers were judged to be the most efficient in the terms of the best phage recovery and stability when compared to anionic or cationic vehicles.

#### 2.2.3 Use accurate dosing devices

While skin phage administration is less common compared to systemic phage administration (Plymoth et al., 2025; Yan et al., 2021), the developments in semi-solid formulations and sophisticated delivery systems reveal a considerable potential for accurate dosing and the successful treatment of local infections. To obtain the best therapeutic results, it is necessary to keep high phage titres and to have a controlled release from the delivery system. The main factors determining success are phage concentration, time of application, and the selection of the pharmaceutical vehicle (Morozova et al., 2018). Semi-solid carriers like gels and creams have been reported to be stable and active *in vitro*, thus showing good potential for phage therapy (Brown et al., 2017; Chang et al., 2020), while innovative platforms such as hydrogel matrices, lipid vesicles, and nanocarriers can be used for enhanced topical targeting (Yao et al., 2025). Metered-dose devices are instrumental in ensuring that the correct application and dosing are achieved (Liu et al., 2016). Techniques such as the Franz diffusion cell used for validation of release profiles demonstrate that phages capable of infection are released from their carriers (Chang et al., 2020). Particle-based systems, primarily nanoparticles, thus, offer even more possibilities for customized, prolonged phage release to facilitate the maintenance of therapeutic concentrations at the infection site (Cobb et al., 2019).

#### 2.2.4 Monitor stability and efficacy

Phages therapeutic efficacy and stability are very sensitive to environmental and formulation factors. The changes in pH, ionic composition, temperature, light, and even the type of packaging material can affect their viability (Samir, 2023; Shlezinger et al., 2019; Wortelboer and Herrema, 2024). To avoid a situation where the activity is lost during production, storage, or use, an in-use stability testing program should be implemented (Duyvejonck et al., 2021; Mary et al., 2024). It has been chosen to conduct spray-drying, freeze-drying, extrusion, and polymer-based encapsulation processes to improve the stability (Samir, 2023; Shlezinger et al., 2019). For long-term storage, the production of the pharmaceutical formulation should be capable of retaining the phage titer for a long time (Malik et al., 2017; Samir, 2023). In addition to this, the formulation also greatly influences the efficiency of the therapy. One of the methods like liposomal encapsulation or powder conversion can serve as a protection for the phage and enhance the targeted delivery to the infection sites (Mulani et al., 2019). If the drugs are topical, the delivery devices should guarantee the deep infiltration of the infected tissues or the biofilm layers in order to achieve high antibacterial and antibiofilm activity of the phages (Xue et al., 2024). Filamentous bacteriophages can also be engineered to serve as targeted nanomedicines with favorable pharmacokinetics. Vaks and Benhar (2011) developed such a system by constructing a phage-based drug carrier. Specificity was achieved by displaying a targeting antibody on the phage tip, while a large antibiotic payload was chemically conjugated to the phage coat. A key innovation was the use of an aminoglycoside linker, which enhanced drug solubility and allowed for controlled release at target sites like bacteria or tumor cells. In a murine model, these drug-carrying phages loaded with chloramphenicol via the aminoglycoside linker proved non-toxic and exhibited reduced immunogenicity compared to native phages. Moreover, they also demonstrated a slower blood clearance rate, a beneficial trait for therapy. The study concludes that aminoglycoside linkers are effective not only for improving drug solubility but also for enhancing the overall safety profile of targeted phage nanomedicines.

## 3 Influence of bacterial load and biofilm maturity on dose selection in local phage therapy

Phage therapy’s effectiveness to kill bacteria that can form biofilms is dependent on two factors mainly: the number of bacteria and the age of the biofilm. On the one hand, a large bacterial load allows phage to replicate, which is frequently required for the treatment to be efficient. On the other hand, mature biofilms can hinder phage penetration due to a thick extracellular matrix, thus making the elimination of chronic infections more difficult (Chang et al., 2022a). As a matter of fact, mature biofilms may even reach equilibrium with phages and weaken the effect of additional doses, hence, the use of phages at an early stage or even prophylactically is usually more efficient in preventing the formation of biofilms (Alves et al., 2018). Thus, the first dose and local phage dynamics at the infection site are very important: phage activity can be elevated by high bacterial loads which in turn can result in great decreases of pathogen numbers (Shahdadi et al., 2023; Theuretzbacher and Piddock, 2019). The presence of biofilm usually entails the repetition or continuation of administration to ensure that the therapeutic effect is sustained via “lysis from without” mechanisms (Chan and Abedon, 2012). Biofilm in bovine mastitis is a major reason for chronic, hard-to-treat udder infections. A study by Banar et al. (2025) evaluated a novel bacteriophage cocktail as a potential alternative to antibiotics for treating bovine mastitis caused by methicillin-resistant *Staphylococcus aureus* (MRSA). The researchers isolated and combined two new phages into a cocktail, which demonstrated an augmented host range and virulence. In a murine mastitis model, the phage cocktail significantly reduced the bacterial burden, showing efficacy comparable to antibiotic treatment. Furthermore, the treatment led to a reduction in key pro-inflammatory cytokines (IL-1β and TNF-α) compared to the untreated control.

### 3.1 Biofilm age and phage efficacy

#### 3.1.1 Early-stage biofilms

Phage intervention during initial biofilm development obtains a large biovolume decrease. As an example, a study similar to that of Sandhu et al. (2025), has been mentioned where 85%-98% biofilm reduction was accomplished by a single phage treatment in the early-stage biofilm produced by *Pseudomonas aeruginosa* (Henriksen et al., 2019). In the experiment reported by (Sandhu et al., 2025) the most notable diminution of *S. aureus* biofilm was recorded that is due to exposure to narrow-spectrum phage at 5x10^7^ PFU/ml.

#### 3.1.2 Mature biofilms

Differentially responsive to treatment, mature biofilms are illustrated by the example of 3-day *Escherichia coli* models, where: phage H-19B, a temperate phage, resulted in almost complete lysogenization; on the other hand, a strictly lytic phage T7 was associated with rapid resistance (Moons et al., 2013). Such opposing results call for the development of specific approaches to get rid of mature biofilms.

### 3.2 Resistance development

Repeated phage monotherapy on mature biofilms that is less responsive to the phage leads to resistance evolution. Biofilms of *Pseudomonas aeruginosa* that were treated several times showed quickly decreased effectiveness due to resistance mechanisms such as modification of the phage receptor and overproduction of alginate for which the mutants were isolated (Martinet et al., 2024). On the other hand, a phage-antibiotic combination therapy acting synergistically is more effective and can also prevent the occurrence of resistance. The sequential administration of phages and then antibiotics resulted in a reduction of the biofilm viability (Kumaran et al., 2018). Soto-Rodriguez et al. (2025) study showed that a phage cocktail provided protection during the most severe acute phase of *Vibrio* infection in shrimp, lowering the virus load and thus early mortality and the shortening of the disease course within the first 12 hours post-infection. Nevertheless, due to the development of phage-resistant strains limiting long-term efficacy, the researchers came to the proposition that the use of phages together with other agents as a synergistic approach would be a safer approach to effective disease control.

### 3.3 Phage dosing strategies

Usually, increased levels of phage lead to more efficient biofilm control (Sandhu et al., 2025). As an example, biofilms that were exposed to higher phage levels (1010 PFU/mL) had reduced bacterial growth significantly in comparison with that of the lower dose (≤104 PFU/mL) (Young et al., 2024). Similarly, additional and consecutive dosing may be intensified. In a dual-species biofilm model, applying phages multiple times (three doses every 8 hours) significantly increased biofilm-killing capacity (Akturk et al., 2023; Sandhu et al., 2025).

### 3.4 Combination therapies

New methods, such as phages loaded with silver nanoparticles, have been demonstrated to have very long antimicrobial activity and substantially enhanced biofilm eradication over long periods of time (Szymczak and Golec, 2024). Using phages in combination with antibiotics, especially in a sequential manner, can significantly contribute to biofilm eradication. The combination of phages with ceftolozane/tazobactam reduced biofilm viability by about 6-log in susceptible *Pseudomonas aeruginosa* strains, as an instance (V. de C. Oliveira et al., 2024).

On the one hand, combining phages with antibiotics or agents that break down biofilms usually results in better biofilm clearance and the resistance to be less likely to occur gradually (Chang et al., 2022a; Gliźniewicz et al., 2025). The order and the ratio of substances used in the co-treatment not only determine the effectiveness and resistance to be controlled but also greatly depend on the stage of infection and maturity of the biofilm, thus dosing regimens should be adjusted accordingly to get the best outcomes (Gliźniewicz et al., 2025; Petrovic Fabijan et al., 2023).

## 4 Pharmacokinetics of topical phage applications

**Figure 2:**
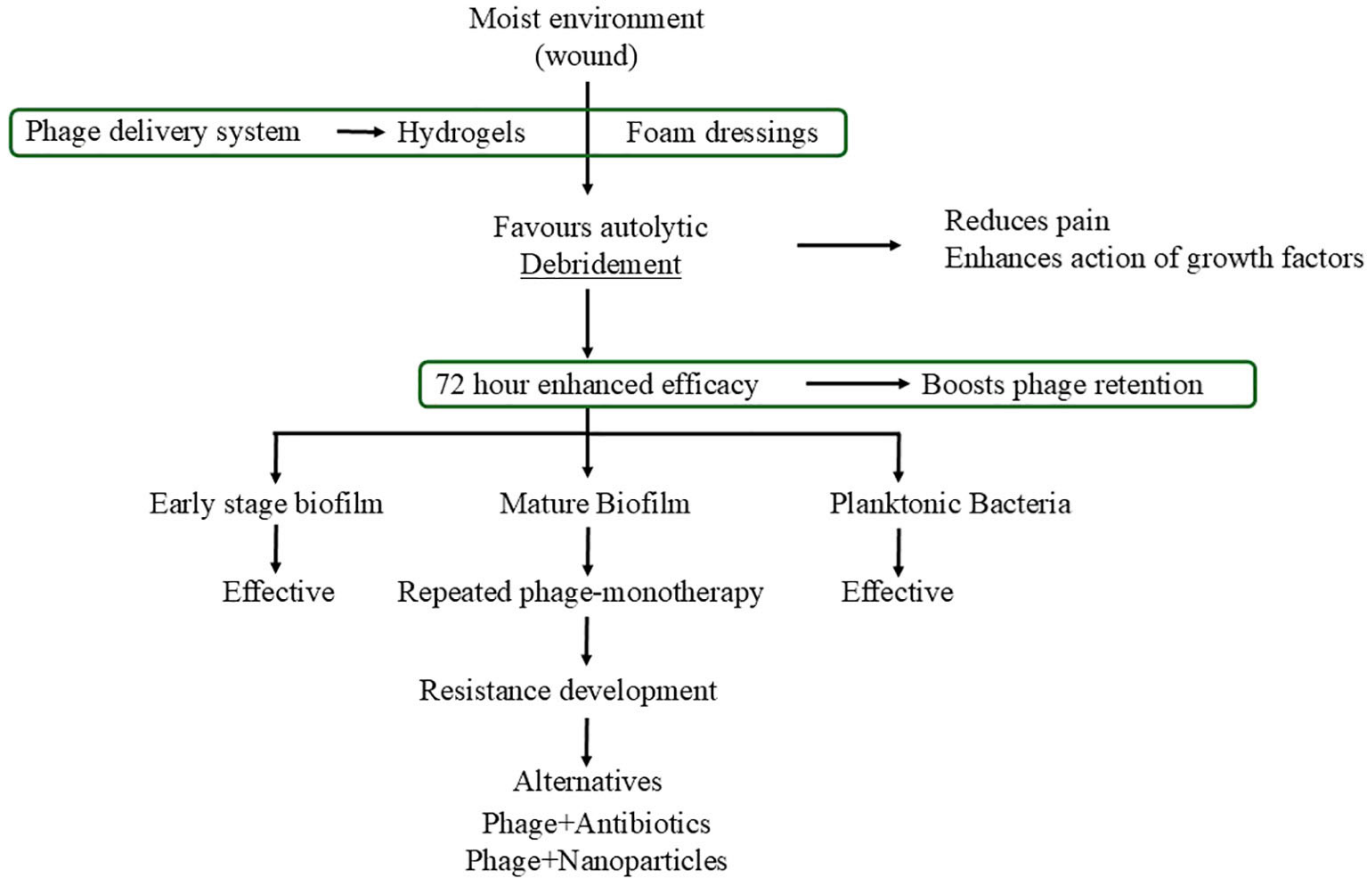
Overview of phage delivery systems in wound management.

Phage administration after wound debridement is estimated to establish a therapeutic window of about 72 hours during which the efficacy of antiseptics and antibiotics against biofilms and planktonic bacteria is significantly higher (Kucisec-Tepes, 2016). In this way, phage application in this timeframe can not only be retained better on the wound surface, but also the therapeutic effectiveness can be increased and for synergetic interactions with other antimicrobial strategies can be enabled. The use of antibiotics in combination with phages, in particular, has been demonstrated to be an extremely powerful tool in the treatment of biofilm-related infections; e.g., *in vitro* studies on *Pseudomonas aeruginosa* biofilm show that simultaneous usage with gentamicin leads to a significant improvement in biofilm eradication, and the highest effects under multi-dose treatment protocols (Akturk et al., 2023). **Figure 2** represents an overview of phage delivery systems used in wound management.

### 4.1 *In Vitro* models

*In vitro* models such as polystyrene microplates and porcine skin explants demonstrate phage effectiveness in drastically lowering biofilm bacterial counts. These setups reveal phage-host interactions and cocktail efficiency, with porcine skin particularly allowing the evaluation of wound surface penetration and retention (Milho et al., 2019). Cell line models are necessary local phage therapy research tools due to their fast generation, low cost, and ease of the experiment. Phages directly applied to mammalian tissues in these systems open the way for mechanistic studies of phage-tissue interactions which are essential for the development of therapy (Williams et al., 2023; Fazilaty, 2022). Regulatory agencies consider such models as *in vivo* surrogates for their first assessment (Garg et al., 2022).

Commonly Used Cell Lines:

1. Caco-2: Frequently used for permeability and pharmacologic assays (Cimbalo et al., 2022; Naiyeju and Bimbo, 2021).
2. RAW 264.7: Utilized in various studies for its ease of use and affordability (Naiyeju and Bimbo, 2021).
3. Other cell lines: HepG2, THP-1, HaCaT, and others are also employed in phage therapy research (Cimbalo et al., 2022; De Souza et al., 2024).

### 4.2 *In Vivo* models

Research by Albac et al. (2019) assessed the *in vivo* effectiveness of a mixture of three bacteriophages versus linezolid in two mouse models of *Staphylococcus aureus* foot infection; non-diabetic and diabetic. A single phage cocktail injection in the hind paw exhibited, in both models, overwhelming antibacterial activity. Though linezolid kept pace with phage therapy in the case of nondiabetic mice, it did not work in diabetic ones. Hence, the findings constitute additional proof that phage therapy could be a viable option for the upcoming preclinical and clinical stages.

The most commonly used *in vivo* models murine systems, wax moth larvae, and zebrafish allow the evaluation of therapeutic efficacy and safety, however, they cannot accurately predict human reaction due to differences between species. Nevertheless, these models are still necessary for studying phage aspects such as tissue diffusivity, bacterial targeting capability, immune interactions, and resistance evolution (Xue et al., 2024). Local phage therapy has proven effective in managing localized bacterial infections, particularly in skin wounds, burns, diabetic conditions, and soft-tissue infections. Preclinical murine studies demonstrate superior infection control with phage formulations compared to standard antimicrobials such as silver nitrate and antibiotics, with combination approaches further improving outcomes in resistant infections like MRSA. Targeted phage delivery has also shown efficacy against multidrug-resistant pathogens, including Acinetobacter baumannii and Klebsiella pneumoniae, highlighting its potential (Kumari et al., 2010; Chhibber et al., 2013; Trigo et al., 2013; Yin et al., 2017; Shlezinger et al., 2019; Jun et al., 2014; Banar et al., 2025). Details are summarised in **Table 2**.

**Table 2:** Preclinical studies evaluating local and topical bacteriophage therapy for site-specific infections.

Infection type	Routes of administration	Dosing regimens	Outcome	Adverse events	Reference
Third degree burn wound infection in BALB/c mice, 6 weeks old, weighing 20–25 ± 5 g with *Klebsiella pneumoniae* B5055	Topical	Phage Kpn5 mixed with hydrogel applied topically at an MOI of 200 on the burn wound site.	Potential to prevent infection in mice compared to multiple silver nitrate and gentamicin treatments.	None	(Kumari et al., 2010)
Diabetes induced BALB/c female mice, 4–6 weeks old weighing 20–25 g infected with MRSA	Topical	The phage MR-10, administered at a dose of 108 PFU/mL (MOI-100).	Combination therapy using bacteriophage and linezolid was more successful in preventing the infection process than antibiotics or phage used alone.	None	(Chhibber et al., 2013)
Foot pad mouse model in eight-week-old female BALB/c mice	Local (subcutaneous)	A single dose of mycobacteriophage D29 containing 8 log_10_ PFU. MPB was given to control (non-treated) mice.	Significantly reduced the number of *M. ulcerans*, increased levels of cytokines (including IFN) and generated a lymphocytic/macrophage-profiled cellular infiltration.	None	(Trigo et al., 2013)
36 six-week-old male BALB/c mice with full-thickness wounds created on the dorsal side infected with drug-resistant *A. baumannii*	Locally (subcutaneous & local administration)	Treatment with the phage Abp1 (5.0 × 10^8^ PFU/mL) once a day for 7 days after infection.	Abp1 is highly stable across temperature and pH ranges and exhibits low lysogeny; locally applied phage significantly reduced wound size compared with systemic or no treatment.	None	(Yin et al., 2017)
Root canal infected model; Wistar rats (250–300 g body weight, 12 weeks old with exposed pulps open to oral cavity	Intramuscular	Phage cocktail with 10^9^ PFU/mL EFDG1 and 10^9^ PFU/mL EFLK1	The phage cocktail showed antibacterial activity and eradicated planktonic *E. faecalis* after 1, 2, 8, 14, 21, and 28 days.	None	(Shlezinger et al., 2019)
*V. parahaemolyticus* CRS 09-17 infection mouse model	Oral	Single dose of phage treatment (2.0 × 10^8^ PFU per mouse)	The phage-treated mice appeared to be only slightly ill and were protected up to 84% (oral) from the lethal infection induced by CRS 09-17 (2.0 × 10^7^ CFU)	None	(Jun et al., 2014)
MRSA Mastitis model, female BALB/c mice	local	1.0 × 10^10^ PFU/ml	The phage suspension significantly reduced bacterial burden in a BALB/c masti- tis model. Moreover, its administration led to decreased concentrations of IL-1β and TNF-α.	None	(Banar et al., 2025)

The effectiveness and safety of phage therapy *in vivo* have been demonstrated by a various number of models which differs in their advantages and limitations:

- Rodent Models: Murine systems provide a very close-to-human physiological scenario for the recreation of human infections and thus, give strong evidences of the therapeutic efficacy (Gómez-Ochoa et al., 2022). In preclinical pneumonia models, Delattre et al. (2022) found that phage delivery directly into the trachea led to about 5,000 times higher exposure of the lungs than the phage delivery through the blood. Also, by applying a semi-mechanistic mathematical model, they demonstrated that the bacterial clearance and host survival become efficient only through a continuous interaction between phages, bacteria, and the innate immune response.
- Wax Moth Larvae and Zebrafish: Wax moth larvae and zebrafish provide easy and fast screening but very often they cannot anticipate human responses accurately due to basic biological differences between species (Xue et al., 2024).
- Non-*In Vivo* Models: There are other options like organ-on-chips, 2D and 3D cell cultures, organoids, and patient-derived xenografts that can overcome some limitations of conventional *in vivo* models. As an example, organoids simulate the cellular complexity of organs but do not have the natural microbial community (Xue et al., 2024).
- Clinical Trials: Though not yet extensively approved, a few studies have advanced to the clinical trial stage in the EU, showing good outcomes in human patients, e.g., the treatment of burn wound infections (Gundogdu et al., 2016).

It is tough to apply the results of preclinical models directly to humans due to differences of biology between species and variations in the design of studies. The availability of excellent preclinical data from both *in vitro* and *in vivo* models is a prerequisite for the phage therapy license (Yang, 2021). In this context, the emerging clinical evidence summarized in **Table 3** complements preclinical findings by demonstrating the feasibility and applicability of local and adjunctive bacteriophage therapy across diverse human infectious indications.

**Table 3:** Summary of published clinical studies evaluating local and adjunctive bacteriophage therapy across diverse clinical indications.

Indication/Setting	Local phage therapy	Other routes used	Duration of treatment	Concurrent antibiotics
Orthopedic infections (Young et al., 2024)	35% of 137 patients	IV/intraarticular combos	1–2 weeks (31.4%), >2 weeks (24.1%)	Not specified
Non-resolving *Pseudomonas aeruginosa* infections (Onallah et al., 2023)	16 patients	Intravenous, topical	Minimum 8 days	Yes
Chronic rhinosinusitis (pediatric) (Krivopalov et al., 2023)	20	Intranasal (sinus rinse)	None	14 days
Cystic fibrosis (*P. aeruginosa*) (Chan et al., 2025)	12	Nebulized/inhaled	IV (in some cases)	7–14 days
Burn wound infections (Tulupov et al., 2023)	30	Topical	Systemic (IV)	7 days
Periprosthetic joint infection (Cammuso et al., 2025)	1	Intra-articular, topical	IV	14 days
Ventilator-associated pneumonia (Navaeifar et al., 2025)	40	Inhaled (nebulized)	Systemic (IV)	7–10 days
Chronic wound (mixed infections) (Beschastnov et al., 2024)	25 8	Topical	None	7 days
Compassionate use (various) (Khatami et al., 2022)	50–100	Local (varied), IV	Systemic	8 days–52 weeks
Chronic osteomyelitis (Onsea et al., 2019)	4 patients	1 intravenous	1 week to 3.5 years (follow-up)	Not specified
Aortic graft/LVAD infections (Chan et al., 2018)	2 patients	1 intravenous	7–9 months (follow-up)	yes

### 4.3 Best dressing type for local phage therapy

The selection of wound dressing is a key factor determining the success of local phage therapy. Those dressings which keep the wound in a moist environment are usually advocated since they lead to better healing results (Nuutila and Eriksson, 2021; Powers et al., 2013; Shimada, 2018; Triller et al., 2013). It is particularly the case of hydrogels and foams that can regulate the therapeutic release through controlled phage therapy. Such systems not only retain phage viability but also facilitate uninterrupted delivery (Herskovitz et al., 2017; Mary et al., 2024; Nuutila and Eriksson, 2021). For instance, hydrogels are considered the most effective carrier agents due to the fact that they absorb water and enable the gradual release of phages, thus, they help the wound healing process and provide a sustained local activity (Mary et al., 2024; Zyman et al., 2022).

### 4.4 Effect of moisture

Keeping the correct moisture level is crucial for wound healing and also for the effectiveness of phage therapy to be at its highest level. A moist environment enables the body’s own enzymes to break down dead tissue, it is less painful and it supports the effect of growth factors and other healing mediators (Ellis, 2015; Nuutila and Eriksson, 2021; Ousey et al., 2016). Overhydration may cause maceration and secondary infection, whereas underhydration results in tissue drying and the prolongation of the healing process (Ellis, 2015; Ousey et al., 2016). Thus, hydrogels and foam dressings that assist in moisture regulation are highly recommended as phage delivery devices (Nuutila and Eriksson, 2021; Powers et al., 2013; Shimada, 2018).

### 4.5 Local degradation/inactivation of phage in wound environments (Biofilm)

Biofilms present in wounds may make bacteria less vulnerable to phage attack and even facilitate the development of resistance (Kim and Kim, 2019; Milho et al., 2019; Namonyo et al., 2023). The phages which produce depolymerases are capable of tearing down the biofilm matrix thus, bacteria become more accessible (D. P. Pires et al., 2016). The use of phages with antibiotics or a natural agent like honey has also been reported to be more effective in antibiofilm effects (A. Oliveira et al., 2017; Stachler et al., 2021). On the other hand, factors in the environment like pH and moisture may also influence the stability of phages; however, proper formulations such as cetomacrogol-based creams can ensure the continuous release of phages and thus help in maintaining their activity even in hard-to-treat wounds (Mary et al., 2024).

## 5 Pharmacodynamics in wound settings

Phages can do the most damage to planktonic bacteria that are free-floating because they can easily find hosts and multiply rapidly. On the other hand, biofilms that have the tightly packed EPS matrix and cells that are slow-metabolizing hinder phage penetration and lower their sensitivity, therefore, treatments need to be resorted to such as enzymatic EPS degradation and very high MOIs to be effective.

### 5.1 Efficiency in planktonic vs biofilm states

Phages infect planktonic bacteria that are fast-growing and easily accessible, a situation that makes lysis in planktonic cultures much faster than in biofilms (Adnan et al., 2020; Li et al., 2025; Nouraldin et al., 2016). In contrast, biofilms bring on physical and metabolic challenges: their intricate extracellular polymeric substance (EPS) matrix hampers phage diffusion, and the slower metabolism of biofilm cells makes them less vulnerable to infection (Nouraldin et al., 2016; D. Pires et al., 2017; Vukotic et al., 2020). Phages inside biofilms are able to move along water channels to reach and copy themselves at a local site (Wasfi et al., 2020). Although a low MOI is usually enough to eliminate a planktonic population, the use of biofilms necessitates much higher MOIs in order to increase the probability of receptor binding and to bring about “lysis from without” in the metabolically active subpopulations (Vukotic et al., 2020).

### 5.2 Host–pathogen–phage dynamics in local phage therapy

The effectiveness of phage therapy depends on the triple interplay among the phages used for therapy, the pathogens that are targeted, and the host microbiome. Factors that matter a lot are the specificity between phage and host, the different ways resistance can be evolved, as well as exact models of these interactions. Since phages attack specific bacterial hosts, they can be instrumental in the conservation of microbial diversity as they do not affect non-target species (Ye et al., 2019). The introduction of strong, very lytic phages can make the pathogen load go down in a short time even before resistance gets developed (Castledine and Buckling, 2024), however, a heavy or repeated phage application can select for resistant subpopulations, thus creating a great therapeutic problem (Roach et al., 2017). The result mostly depends on the resistance–infectivity trade-off situation: if resistance does not lead to the reduction of pathogen fitness or virulence, phage therapy may not work (Merikanto et al., 2018). To solve the contradictions between clinical goals and biological limitations, it is necessary to get more mechanistic understanding of phage-bacteria-immune interactions (Roach et al., 2017). To be accurate in predictions, there should be a set of very advanced preclinical models, which should include organoids and patient-derived xenografts though the current systems often are not able to correctly estimate human responses (Xue et al., 2024).

## 6 Conclusion and future perspective

Topical phage therapy is a very potential method to treat infections, but its achievement is highly dependent on whether the dosing and formulation are tailored appropriately. In order to be most effective, doctors have to ensure that therapeutic phage concentrations are maintained, the correct multiplicity of infection (MOI) is chosen, and stable delivery systems such as hydrogels, cetomacrogol creams, or metered sprays are employed. These agents bind the phages to the wound site and facilitate the release of the phages. Besides that, the most important factors to success are applying phages early against biofilms that are forming, combining them with antibiotics or depolymerases, and checking for resistance. At last, the steps from promise to clinical practice include standardized pharmacokinetic/pharmacodynamic (PK/PD) studies, stability tests, and clinical trials. Coordinated research and optimized delivery can make topical phage therapy a dependable weapon against resistant and biofilm-associated infections, thus providing a safer, more targeted means of infection control.

## 7 Expert opinion

Topical phage therapy has the potential to be the most significant factor in changing the way we handle local, antibiotic-resistant and biofilm-related infections, especially in wound care. However, its first effect in reality will be the solving of various practical, regulatory and technical bottlenecks.

1. Impact on real-world outcomes and feasibility of implementation.

By the use of topical phage regimens, clinical outcomes could be improved to a great extent. This could be achieved by the targeted clearance of bacteria with very minor disruption of the rest of the microbiome, thus the need for systemic antibiotics would be reduced and the rates of chronic wound colonization and recurrent infection would be lowered (Mukhopadhyay et al., 2024). Economically, successful phage products (e.g., phage-impregnated hydrogels or metered sprays) could reduce hospital days, dressing changes, and antibiotic costs, particularly in high-burden settings (burn units, diabetic foot clinics). Realistic clinical implementation is achievable first in niche settings compassionate use, specialized wound centers, and adjunct protocols where clinicians already accept nonstandard antimicrobials (Wang et al., 2024b). Wider adoption is prevented today by lack of standardized dosing/PK-PD frameworks, variable product stability and manufacturing practices, sparse randomized controlled trial (RCT) evidence, and unclear regulatory pathways for individualized biological therapeutics. Phage therapy will be mostly a trial issue until the authorities give clear and scalable ways of approval and the producers provide reproducible and stable formulations under GMP (Cui et al., 2024; Kim et al., 2025).

2. Key areas for improvement and how to solve limitations.

Several practical shortcomings have been listed which need to be resolved: (a) Standardization - standard units for titers, MOI, stability tests, release kinetics, and PK endpoints on which to base different research articles are extremely necessary; learned societies and funders should require minimal datasets for preclinical and clinical reports as a condition of publication. (b) Formulation & stability - better encapsulation, lyophililization, and protective excipients will lessen the need for cold-chain and thus allow for off-the-shelf topical products; industry-academic partnerships should focus on scalable formulation engineering to open the way for more industrial processes. (c) Dosing science - the establishment of robust PK/PD models for topical compartments (wound surface, biofilm microenvironments) and the availability of validated biomarkers of phage activity (local titer measurements, rapid host-pathogen burden assays) would help in dose and schedule determination. (d) Resistance and immune neutralization - routine use of multi-phage cocktails, rotating banks of phages, and combination strategies (antibiotics, depolymerases) can mitigate resistance; surveillance frameworks should be established to detect resistance early. (e) Regulatory & manufacturing - harmonized regulatory guidance on quality attributes, potency assays and acceptable manufacturing variation will be decisive; public funding to de-risk manufacturing scale-up for academic phage libraries could accelerate translation (Ikpe et al., 2024; Palma and Qi, 2024; Suleman et al., 2024; Wdowiak et al., 2022).

3. Potential value of further research and endpoints.

Further research can deliver definitive, practice-changing outcomes if it focuses on translational endpoints: clinically meaningful wound healing metrics, infection recurrence, antibiotic consumption, cost-effectiveness, and safety (including immunogenicity and microbiome effects) (Wang et al., 2024b). There is unlikely to be a single ultimate “end-point” for phage therapy - rather, progress will be incremental and contextual: approved topical products for specific indications (e.g., infected burn wounds, chronic diabetic ulcers) and validated adjunctive regimens that reduce antibiotic use. Research should prioritize well-designed RCTs and pragmatic trials in high-need populations, alongside mechanistic PK/PD studies that permit regulatory acceptance (Pinto et al., 2020; Young et al., 2023).

4. Relative promise of this area vs other directions.

Topical phage therapy is among the most promising near-term translational routes for phages because local delivery avoids many systemic hurdles (immune neutralization, biodistribution). However, complementary areas - engineered bacteriophages (to deliver depolymerases or narrow host-range modifiers), phage-derived enzymes, and synthetic antibiofilm materials - also deserve investment. The most productive future will likely be combinatorial: phages integrated into engineered dressings, combined with small-molecule or enzymatic adjuvants, monitored by rapid diagnostics (Lin et al., 2025; Palma and Qi, 2024; Zyman et al., 2022).

5. How the field will evolve over 5–10 years.

Within five to ten years we expect a layered evolution rather than a single revolution. Early wins will be niche, evidence-backed topical products used in specialized centers (e.g., phage-impregnated hydrogel dressings, metered-dose topical sprays) and formal incorporation of phage options into wound-care formularies where trials show benefit. Standardized PK/PD reporting and stability testing will become routine, enabling regulators to define clearer approval pathways. Technological gains better encapsulation, cold-chain-free formulations, point-of-care susceptibility tests and smart dressings that modulate release - will broaden accessibility and lower costs. What may be lost is the “one-size-fits-all” mentality: clinicians will increasingly accept personalized or adaptive regimens tailored by rapid diagnostics rather than fixed antibiotic courses (Alberts et al., 2025; Yadav et al., 2024).

## 8 Five-year speculative viewpoint

Five years from now the field will likely have achieved modest but concrete clinical traction: one or more commercially manufactured topical phage products (e.g., hydrogel or cream) approved for narrow indications, a handful of positive multicenter pragmatic trials showing reductions in antibiotic use and improved wound outcomes, and accepted minimum reporting standards for phage dosing and PK/PD. Clinics managing complex wounds will incorporate phage susceptibility testing into their diagnostic workflows and use metered phage dressings as adjuncts to debridement and antibiotics. Regulatory agencies will offer clearer guidance for topical phages, but global, routine use in general practice will still hinge on continued scale-up, cost reductions, and broader RCT evidence. Overall, topical phage therapy will move from intriguing experimental therapy toward a pragmatic, evidence-informed tool in the wound-care armamentarium - complementary to, not replacing, antibiotics.
